# Osteoprotective Effects in Postmenopausal Osteoporosis Rat Model: Oral Tocotrienol vs. Intraosseous Injection of Tocotrienol-Poly Lactic-Co-Glycolic Acid Combination

**DOI:** 10.3389/fphar.2021.706747

**Published:** 2021-11-18

**Authors:** Nurul ‘Izzah Ibrahim, Hasnul ‘Iffah Mohd Noor, Ahmad Naqib Shuid, Sharlina Mohamad, Mohd Maaruf Abdul Malik, Putri Ayu Jayusman, Ahmad Nazrun Shuid, Isa Naina Mohamed

**Affiliations:** ^1^ Department of Pharmacology, Faculty of Medicine, Universiti Kebangsaan Malaysia Medical Centre, Kuala Lumpur, Malaysia; ^2^ Advanced Medical & Dental Institute (AMDI), Universiti Sains Malaysia, Kepala Batas, Malaysia; ^3^ Centre of Preclinical Science Studies, Faculty of Dentistry, Universiti Teknologi MARA, Sungai Buloh Campus, Jalan Hospital, Sungai Buloh, Malaysia; ^4^ Department of Pharmacology, Faculty of Medicine, Universiti Teknologi MARA, Sungai Buloh Campus, Jalan Hospital, Sungai Buloh, Malaysia

**Keywords:** osteoporosis, oxidative stress, tocotrienol, PLGA, intraosseous

## Abstract

Osteoporosis, the most common bone disease, is associated with compromised bone strength and increased risk of fracture. Previous studies have shown that oxidative stress contributes to the progression of osteoporosis. Specifically, for postmenopausal osteoporosis, the reduction in estrogen levels leads to increased oxidative stress in bone remodeling. Tocotrienol, a member of vitamin E that exhibits antioxidant activities, has shown potential as an agent for the treatment of osteoporosis. Most studies on the osteoprotective effects of tocotrienols had used the oral form of tocotrienols, despite their low bioavailability due the lack of transfer proteins and high metabolism in the liver. Several bone studies have utilized tocotrienol combined with a nanocarrier to produce a controlled release of tocotrienol particles into the system. However, the potential of delivering tocotrienol–nanocarrier combination through the intraosseous route has never been explored. In this study, tocotrienol was combined with a nanocarrier, poly lactic-co-glycolic acid (PLGA), and injected intraosseously into the bones of ovariectomized rats to produce targeted and controlled delivery of tocotrienol into the bone microenvironment. This new form of tocotrienol delivery was compared with the conventional oral delivery in terms of their effects on bone parameters. Forty Sprague–Dawley rats were divided into five groups. The first group was sham operated, while other groups were ovariectomized (OVX). Following 2 months, the right tibiae of all the rats were drilled at the metaphysis region to provide access for intraosseous injection. The estrogen group (OVX + ESTO) and tocotrienol group (OVX + TTO) were given daily oral gavages of Premarin (64.5 mg/kg) and annatto-tocotrienol (60 mg/kg), respectively. The locally administered tocotrienol group (OVX + TTL) was given a single intraosseous injection of tocotrienol–PLGA combination. After 8 weeks of treatment, both OVX + TTO and OVX + TTL groups have significantly lower bone markers and higher bone mineral content than the OVX group. In terms of bone microarchitecture, both groups demonstrated significantly higher trabecular separation and connectivity density than the OVX group (*p* < 0.05). Both groups also showed improvement in bone strength by the significantly higher stress, strain, stiffness, and Young’s modulus parameters. In conclusion, daily oral tocotrienol and one-time intraosseous injection of tocotrienol–PLGA combination were equally effective in offering protection against ovariectomy-induced bone changes.

## Introduction

Osteoporosis is the most common bone disease that can be characterized by low bone mass, deterioration of bone tissue, and disruption of bone microarchitecture. These characteristics may lead to compromised bone strength and increased risk of fracture (NIH Consensus Development Panel on Osteoporosis Prevention, Diagnosis, and Therapy, 2001; Sözen et al., 2017). Studies have demonstrated that oxidative stress played a major role in the progression of osteoporosis (Cervellati et al., 2014; Ibáñez et al., 2014). Oxidative stress may promote differentiation of osteoclasts and bone resorption, which would eventually cause impairment of the musculoskeletal system (Baek et al., 2010; Yang et al., 2013). Oxidative stress, which can be described as a disturbance in the balance between reactive oxygen species (ROS) and antioxidants, could be overcome by antioxidants. In general, the human body possesses its own in-built or endogenous antioxidant system, which maintains the balance between ROS production and antioxidants. However, when the endogenous antioxidant system malfunctions, external antioxidants could be administered to reduce the cumulative adverse effects of oxidative stress (Pohl and Kong Thoo Lin, 2018). In the case of post-menopausal osteoporosis, marked reduction in estrogen levels has been associated with increased oxidative stress during bone remodeling (Bellanti et al., 2013; Dalal and Agarwal, 2015; Domazetovic et al., 2017). Several studies have found correlations between antioxidant levels and bone metabolism. A marked decrease in plasma antioxidants was observed in both aged or osteoporotic rats or human subjects (Maggio et al., 2003; Almeida et al., 2007; Sendur et al., 2009). It was shown that intake of antioxidants such as vitamin C and E, N-acetyl-cysteine, and lipoic acid have beneficial effects in individuals with osteoporosis (Sanders et al., 2007; Mainini et al., 2012; Kim et al., 2016). Antioxidants promoted bone health by suppressing inflammation, inhibiting osteocyte apoptosis, and increasing osteoblast activity, thus, preventing oxidative cell damage and bone cell loss (Domazetovic et al., 2017). Antioxidants can be obtained from synthetic (chemically synthesized) or naturally occurring sources. Plant secondary metabolites, such as terpenoids and phenolic compounds, are natural sources of antioxidants (Swallah et al., 2020). Synthetic antioxidants may carry toxicity risks compared with natural sources, which are much safer (Carocho and Ferreira, 2013).

Tocotrienol is a member of vitamin E, which can be naturally found in palm oil, annatto, and rice bran oil (Meganathan and Fu, 2016). Tocotrienol derived from annatto bean is unique because it contains the most active type of tocotrienols, which are delta-tocotrienol (90%) and gamma-tocotrienol (10%) (Yong et al., 2014). When compared with tocopherols, another member of vitamin E, tocotrienol possesses other medicinal properties including anticancer, anticholesterol, and neuroprotection (Sen et al., 2006). Both homologs of tocopherol and tocotrienol are named as α, β, γ, and δ depending on the position and number of the methyl substitutions on the aromatic side of the chromanol ring (Seppanen et al., 2010). Tocotrienol exhibited more potent antioxidant properties than α-tocopherol due to the unsaturated side chain that permits better penetration into tissues (Serbinova et al., 1991; Serbinova and Packer, 1994). Besides, tocotrienol demonstrated superior antioxidant and free radical scavenging activities compared with tocopherol due to their better distribution in fatty layers of cell membrane (Suzuki et al., 1993). Oral supplementations of tocotrienol were able to improve oxidative stress and promote antioxidant enzyme activities in the bones of ovariectomized rats, a postmenopausal osteoporosis model (As et al., 2008; Maniam et al., 2008). Additionally, an *in vitro* study showed that γ-tocotrienol attenuated oxidative damage on primary osteoblast culture by preserving the antioxidant enzyme activities in the bone cells (Nizar et al., 2011; Abd Manan et al., 2012). Annatto tocotrienol enhanced gene expressions related to bone formation and osteoblast activity in testosterone-deficient osteoporotic rat model (Chin and Ima-Nirwana, 2014). Several studies have shown that daily oral tocotrienol intake protected against ovariectomy-induced bone changes in rat models (Mohamad et al., 2012; Soelaiman et al., 2012; Muhammad et al., 2013; Chin et al., 2017). All these studies suggested that tocotrienol protected the bone by increasing osteoblast number, mineral deposition, and bone formation, as well as reducing osteoclast number to prevent bone resorption, erosion, and degeneration of bone mineral density and microarchitecture (Musa, 2021). However, tocotrienol was associated with limited oral bioavailability as they were not recognized by the alpha-tocopherol transfer protein for intake into the body (Packer et al., 2001). Tocotrienol needs to be given orally at high doses to overcome hepatic metabolism and is widely distributed in the body. Thus, only a minor fraction of tocotrienol would reach the bone to exert its effects. It would be beneficial to deliver tocotrienol directly to the bone, rather than indirectly via the oral route.

Controlled drug delivery (CDD) *via* polymeric, targeted, and active release systems extends a drug half-life and improves bioavailability by providing protection against premature degradation. Additionally, the system offers prolonged, sustained, or intermittent release of treatment agent at therapeutic doses to more effectively treat diseases such as osteoporosis and associated fractures (Asafo-Adjei et al., 2016). CDD could also solve problems related to patient noncompliance and systemic adverse effects (Asafo-Adjei et al., 2016). Synthetic and biodegradable polymers, which are commonly used as carriers in drug delivery systems include poly(glycolic acid), poly(lactic acid), poly(D,L-lactide-co-glycolide), poly(caprolactone), and polydioxanone. A formulation that conjugates polymers and tocotrienol could improve its efficacy by controlling the rate, time, and place of release of tocotrineol in the body (Sung and Kim, 2020). A combination of tocotrienol and polymer could be locally injected inside the bone via intraosseous (IO) injection. This is a rapid and safe method for acquiring parenteral access in patients with difficult venous access, in coma or who are immobile (Petitpas et al., 2016). Although IO injection is superior in many clinical situations, it is underutilized, as it is an invasive procedure and require skills to perform the injection (Dornhofer, 2021). In terms of beneficial application to bone, IO injection can be given directly to the vulnerable-to-fracture bone, require lower dose of treatment agent compared with oral dose, and less systemic exposure of the drug to other organs. In addition, IO injection could be an effective method of delivery as bones are highly vascularized, allowing better absorption of treatment agent (Taylor, 2020). Successful use of different agents at different IO injection sites have been reported such as prostaglandin E2 on tibial metaphysis (Yang et al., 1993), fibroblast growth factor on iliac crest (Nakamura et al., 1998), and bone morphogenic protein-2 on the proximal femur (Seeherman et al., 2006). All these studies demonstrated positive outcomes of bone formation and strength.

To the best of our knowledge, no bone studies have been conducted on tocotrienol combined with polymer, given via IO injection. Therefore, this study was designed to determine the effects of tocotrienol–PLGA combination on the tibial bone of postmenopausal osteoporosis rat model. The osteoprotective effects of one-time IO injection of the tocotrienol–PLGA combination were compared to daily oral tocotrienol in the ovariectomized rat model.

## Materials and Methods

### Animals and Surgical Procedure

A total of 40 3-month-old Sprague–Dawley rats weighing 200–250 g were used in this study. The rats were purchased from the Laboratory Animal Resources Unit, Faculty of Medicine, Universiti Kebangsaan Malaysia (UKM). They were kept at two per cage under 12-h light–dark cycle and given tap water *ad libitum*.

Following 1 week of acclimatization, the rats were assigned randomly into five groups with eight rats per group. The first group was sham operated for surgical stress simulations, while the other four groups were ovariectomized (OVX). All surgeries were carried out under anesthesia with intramuscular injection (0.1 ml/100 g body weight) of ketamine and xylazine at a 1:1 ratio. First, the rat abdomen was shaved with an electric shaver and sterilized with 70% alcohol. The skin of the lower middle part of the abdomen was vertically incised at approximately 3 cm using a scalpel. For the ovariectomized group of rats, the fallopian tubes adjacent to the ovaries were cut to remove the ovaries. Abdominal wall and skin were sutured, and iodine solution was applied. Enrofloxacin 5% (Baytril®) was injected intramuscularly at a dose of 0.1 ml/100 g body weight. For the sham-operated group, the above procedures were also performed, but the ovaries were not removed. Following the procedure, the rats were left untreated for 2 months to allow for osteoporosis to develop in the ovariectomized rats. This study has been approved by the Universiti Kebangsaan Malaysia Animal Ethical Committee (FP/FAR/2015/NAZRUN/9-DEC./710-DEC.-2015-OCT.-2017).

### Categorization of Rats and Treatments

The sham-operated (SO) and ovariectomized-control (OVXC) groups were given deionized water via oral gavages. The estrogen group (OVX + ESTO) acted as the positive control group and was given daily oral gavages of Premarin (64.5 mg/kg). The oral tocotrienol group (OVX + TTO) was given daily oral gavages of tocotrienol 60 mg/kg. These four groups also received a one-time intraosseous (IO) injection of PLGA (without tocotrienol) at the proximal metaphysis region of the right tibia. The locally administered tocotrienol group (OVX + TTL) was given a one-time IO injection of tocotrienol at 60 mg/kg combined with PLGA but only received daily oral gavages of deionized water (vehicle only).

### Intraosseous Injection

**FIGURE 1:**
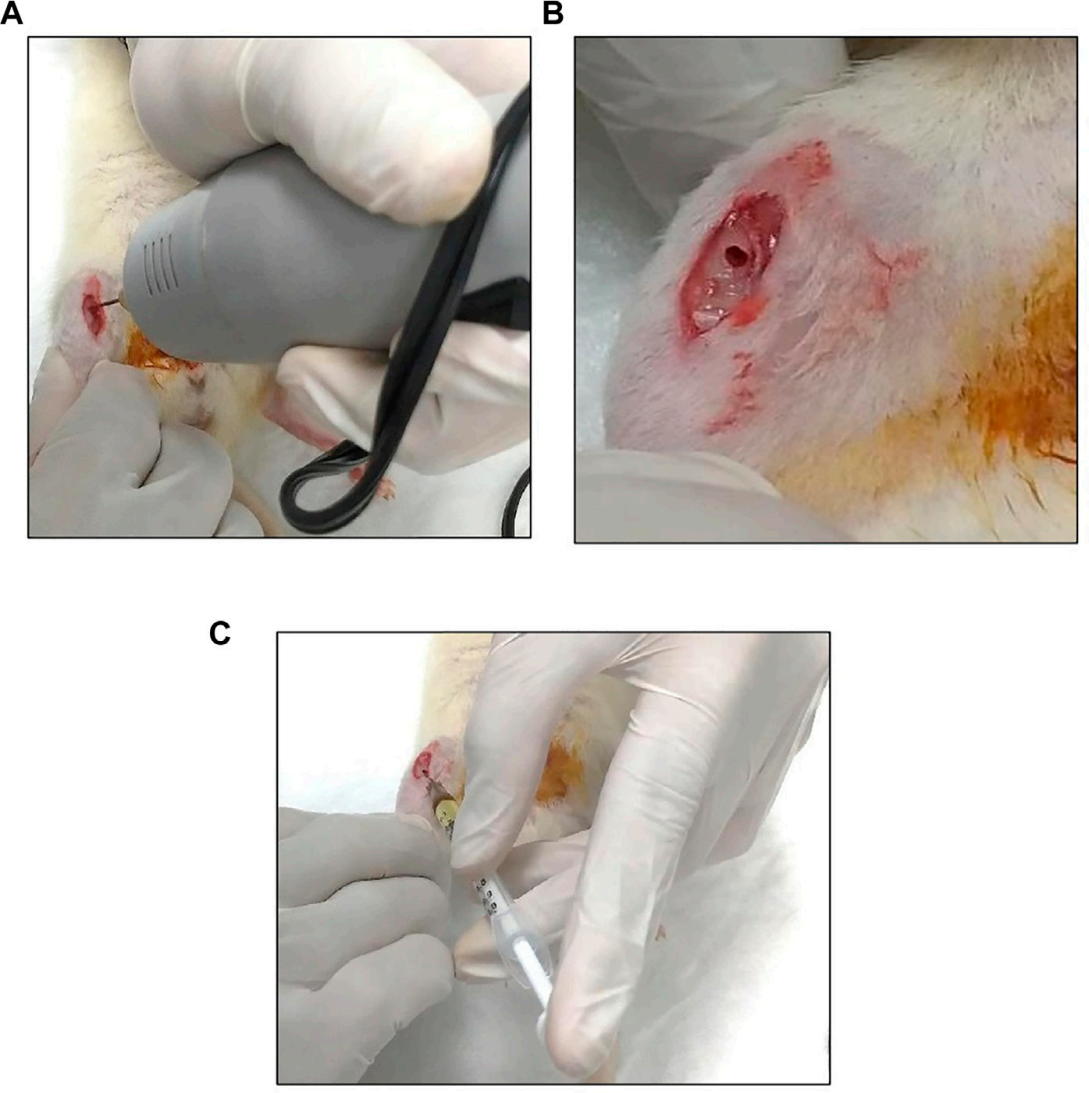
Procedures for intraosseous (IO) injection of the treatment agent to the tibial bone. **(A)** The tibia was drilled using a 1.0-mm drill bit. **(B)** The burr hole at the exposed tibia. **(C)** One time-IO injection of poly lactic-co-glycolic acid (PLGA)/tocotrienol combined with PLGA using a 26-G needle.

The rats were anesthetized with intramuscular injection (0.1 ml/100 g body weight) of ketamine and xylazine at a 1:1 ratio. The right side of the leg was shaved and sterilized with 70% alcohol. An incision was made anterior medially with an extension from the medial femur condyle to the middle of the tibia to obtain access to the right tibia. The proximal tibial was exposed without damaging the flexor and extensor muscle at the tibia. A drill with a drill bit of 1.0-mm size was used to create a burr hole at the flat region of the tibial bone. A needle marked at 3 mm from the tip was used as a guide to ensure a standardized depth of drilling. IO injection was carefully given at a 45° angle to prevent backflow of the agent (Figure 1). The skin at the tibia was then sutured and iodine applied. Enrofloxacin (Baytril^®^) at a dose of 0.1 ml/100 g body weight was injected intramuscularly twice, immediately and after 2 weeks, to prevent infection.

### Preparation of Particles

Annatto tocotrienol (Delta Gold® 70) was supplied by American River Nutrition Inc. (Hadley, MA, United States). Tocotrienol microparticles were prepared according to Urata et al. (1999). Briefly, 200 mg of tocotrienol and 1,000 mg of PLGA were dissolved in 40 ml of dichloromethane and stirred using a magnetic stirrer until they turned into a solution. Subsequently, the solution was cooled to 4°C for 1 h to allow crosslink formation between the tocotrienol and the polymer. The solution was then dispersed in a 0.1 polyvinyl alcohol solution using a magnetic stirrer. The stirring was continued for another 5 h at room temperature to evaporate off the tocotrienol and solvent that did not bind to PLGA. The mixture was filtered using a 0.45-mm filter, followed by filtration using a syringe filter with 0.2-µm pore size to obtain smaller-sized particles. Next, the resultant particles were washed with distilled water, and dried under reduced pressure for 2 days to ensure dryness and evaporation of the remaining solvent. Finally, the particles were collected and suspended in a solution of 0.5% carboxymethyl cellulose and 0.1% Tween 80. The same procedures were performed to prepare the microparticles with polymer only, without the addition of tocotrienol.

### Dual-Energy x-ray Absorptiometry

DEXA is the most commonly used and standard method for diagnosing osteoporosis, as well as assessing osteoporotic fracture risks. It is a precise, stable, and noninvasive scanning method for whole-body bone mass and soft tissue composition measurements. Using phantoms, it is able to verify measurement stability of better than 0.5% change in the accuracy of body composition. It uses a very small dose of ionizing radiation to measure bone mineral density (BMD). It can also be used to produce internal body images to measure bone loss (Shepherd et al., 2017).

Following anesthetization with ketamine and xylazine, the whole body of rats was scanned for a duration of 4 min, while the tibial bone scanning took only 3 min. Prior to the scanning procedure, a Hologic Discovery W DEXA machine (Hologic, Inc., MA, United States) was calibrated using “Small Animal Phantom.” The DEXA analysis was performed using the APEX^TM^ software.

### Bone Biochemical Markers

The level of C-terminal telopeptide of type 1 collagen (CTx) and osteocalcin (OC) were used as reference markers of bone resorption and formation, respectively (Shetty et al., 2016). Fasting blood was collected *via* orbital sinus using capillary pipettes for all the eight rats in each group before and after treatment. Prior to blood collection procedure, the rats were anesthetized with diethyl ether inhalation in a desiccator. After the rats were under anesthesia, a gentle pressure was applied using the thumb on the external jugular vein causing the eyes to bulge. A capillary pipette was inserted ventrolaterally and was rotated a few times until the blood could be withdrawn. Blood was allowed to flow by capillary action into the capillary pipette and collected into a plain tube. Adequate hemostasis was ensured after the procedure. Serum was extracted by centrifugation (3,000 rpm × 10 min) and stored at −80°C until biochemical analyses were performed. Serum CTX and osteocalcin were measured using enzyme-linked immunosorbent assay (ELISA) technique and analyzed using ELISA reader (VERSAmax^TM^, Molecular Devices Corporation, Sunnyvale, CA, United States) and SoftMax^®^ Pro Data Acquisition and Analysis software. The kits used were rat osteocalcin ELISA (IDS Ltd., Tyne and Wear, Boldon Colliery, United Kingdom) and Ratlaps ELISA CTX (IDS Ltd., Tyne and Wear, Boldon Colliery, United Kingdom).

### Microcomputed Tomography

Micro-CT enables high-resolution imaging of bone structure to monitor disease progression and treatment in an animal over a period of time (Shepherd et al., 2017). Micro-CT scanning was performed on the rat right tibia using the μCT system (Skyscan 1076 Bruker, Kontich, Belgium), focusing on the metaphyseal area. The scanning was set at 93-kV x-ray voltage, 100-μA x-ray current, Al 1.0 filter, 0.5° rotation step, and 9 μm pixel resolution. The images were reconstructed using the NRecon software (V1.6.10.4, SkyScan, Kontich, Belgium). The 3D bone parameters for trabecular regions were analyzed using the CTAn software (V1.16.1.0+, SkyScan, Kontich, Belgium). The volume of interest, which was the trabecular region, was set at 1.0 mm from the distal growth plate level, extending toward the proximal end of the tibia (200 slides).

### Bone Biomechanical Testing

The biomechanical test can provide information about the structural and material properties of bone. The three-point bending test is frequently used for long bones, such as the tibia, fibula, and femur. For the test, the span between the lower loaders and the radius of the loading surface curvature should be appropriately selected depending on the animals, species, and sex (Oksztulska-Kolanek et al., 2016). The three-point bending test was performed on the right tibia using an Instron universal material testing machine (Dual Column Model 5560 Series, Norwood, MA, United States) to measure the biomechanical strength of the bone. The machine was equipped with a computer and Bluehill® software version 2. The extrinsic parameters measured include stiffness, load, and displacement, while the intrinsic parameters measured were, stress, strain, and modulus of elasticity. During the test, load was driven down into the center of the bone until it fractures. The load, displacement, stress, and strain parameters were recorded by the software. A graph of load against displacement was plotted, where the rate was proportionate until it reached the ultimate load and the bone fractured. A graph of stress against strain was also plotted, where Young’s modulus could be derived from the curve gradient.

### Statistical analysis

The data were analyzed using the Statistical Package for Social Science software (SPSS version 20.0) and expressed as mean ± SEM. The data were tested for normality using the Kolmogorov–Smirnov test. For normal distribution of data, the statistical tests used were the analysis of variance (ANOVA), followed by *post-hoc* analysis of Tukey’s HSD test. The result was considered significant when the p-value was less than 0.05.

## Results

### Body Weight

**FIGURE 2:**
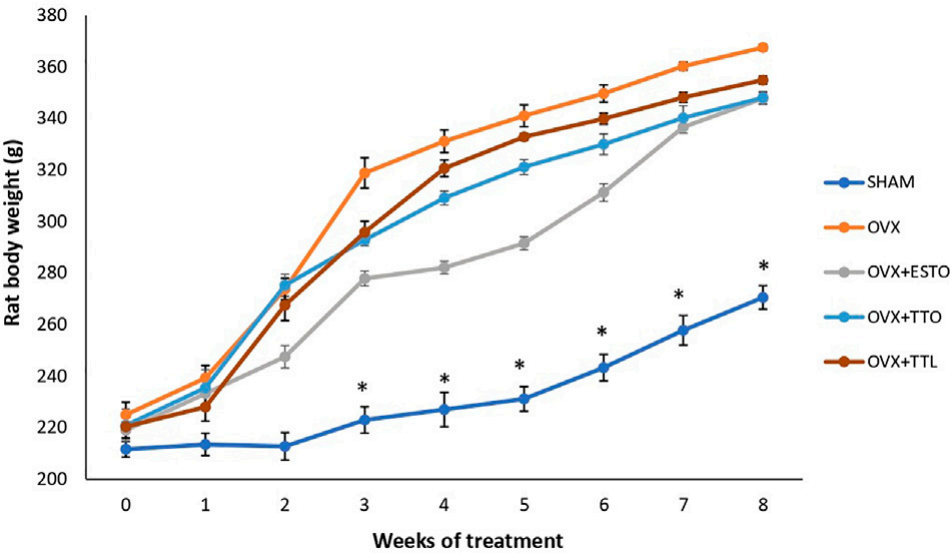
Body weights. SHAM, sham-operated group; OVX, ovariectomized control group; OVX + ESTO, ovariectomized + oral estrogen; OVX + TTO, ovariectomized + daily oral tocotrienol; OVX + TTL, ovariectomized + IO injection of tocotrienol + PLGA. *Significant difference (*p* < 0.05) when compared with the SHAM. The data were expressed as mean ± SEM.

As expected, ovariectomized rats had significantly higher body weights throughout the study compared with sham-operated rats. Administration of estrogen or tocotrienols seemed to attenuate the weight gain induced by ovariectomy but with no significant effects (Figure 2).

### Bone Biochemical Markers

**FIGURE 3:**
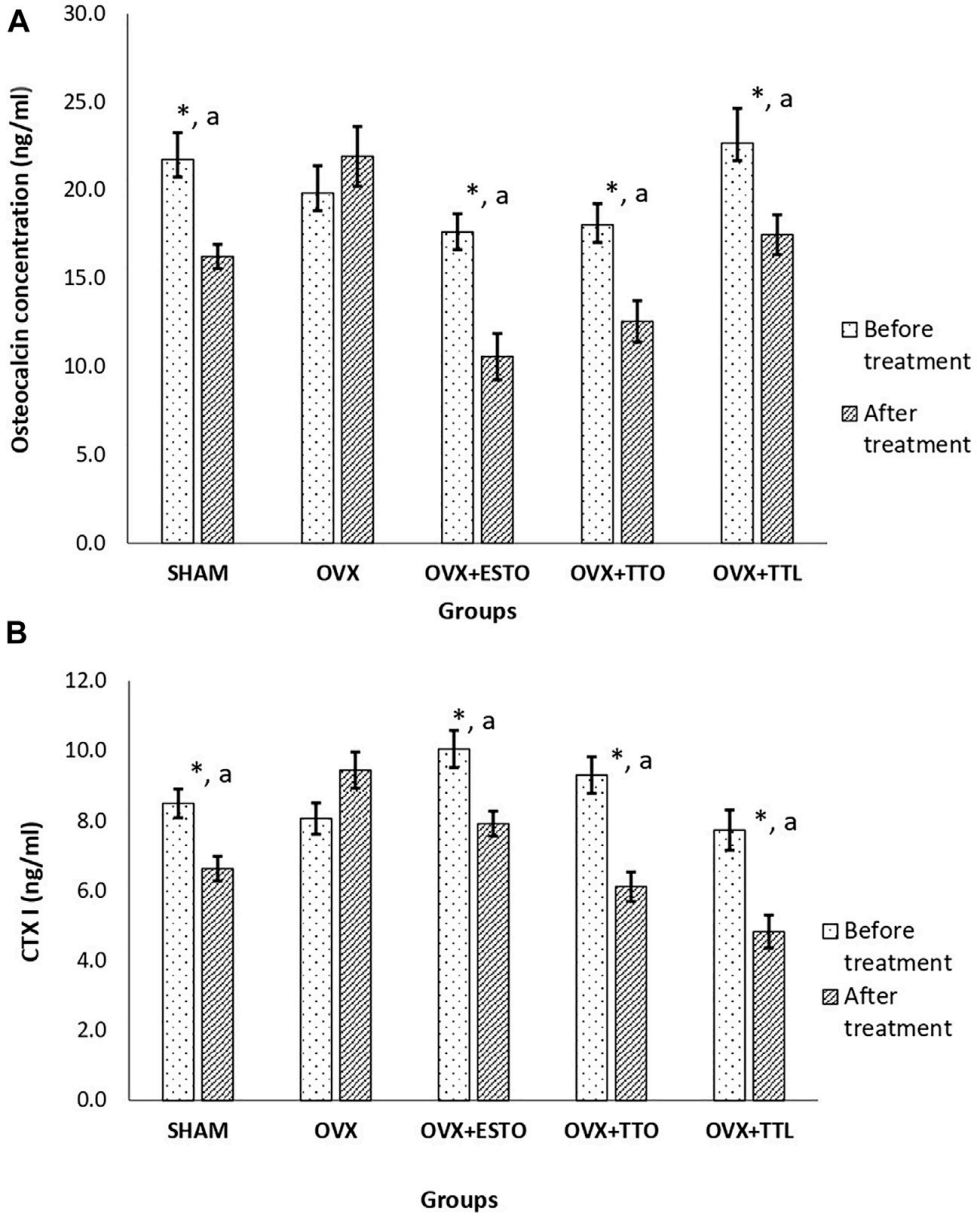
Bone biochemical markers (pre- and posttreatment). **(A)** Pretreatment and posttreatment levels of osteocalcin. **(B)** Pretreatment and posttreatment levels of CTX1. SHAM, sham-operated group; OVX, ovariectomized control group; OVX + ESTO, ovariectomized + oral estrogen; OVX + TTO, ovariectomized + daily oral tocotrienol; OVX + TTL, ovariectomized + IO injection of tocotrienol + PLGA. *Significant difference (*p* < 0.05) when compared before and after treatment ^a^Significant difference (*p* < 0.05) when compared with the ovariectomized control group (OVX). The data are expressed as mean ± SEM.

After 8 weeks of treatment, both the OC (bone formation marker) and CTx (bone resorption marker) levels of ovariectomized-control group (OVX) were maintained at high levels. As for sham-operated group (SHAM), after 8 weeks of treatment, both the bone markers were significantly lower than their pretreatment levels by 25.31% and 21.97% for osteocalcin and CTx, respectively. Oral estrogen (OVX + ESTO) and both forms of tocotrienol (OVX + TTO and OVX + TTL) were able to suppress the bone marker levels until they were significantly lower than their pretreatment levels and the levels of ovariectomized control group (OVX) (Figure 3). The highest percentage of OC reduction was recorded by the positive control group (OVX + ESTO) at 40.08%, followed by the OVX + TTO group at 30.38% and the OVX + TTL group at 22.93%. While for CTx, the OVX + TTL group showed the greatest reduction at 37.52%, followed by the OVX + TTO and OVX + ESTO groups, at 34.31% and 21.25%, respectively (Figure 3).

### Dual-Energy X-Ray Absorptiometry of the Tibial Bone

Bone mineral content is the percentage of bone mineral compared with total body weight. Bone mineral density is the amount of minerals (mostly calcium and phosphorous) contained in a certain volume of bone.

**FIGURE 4:**
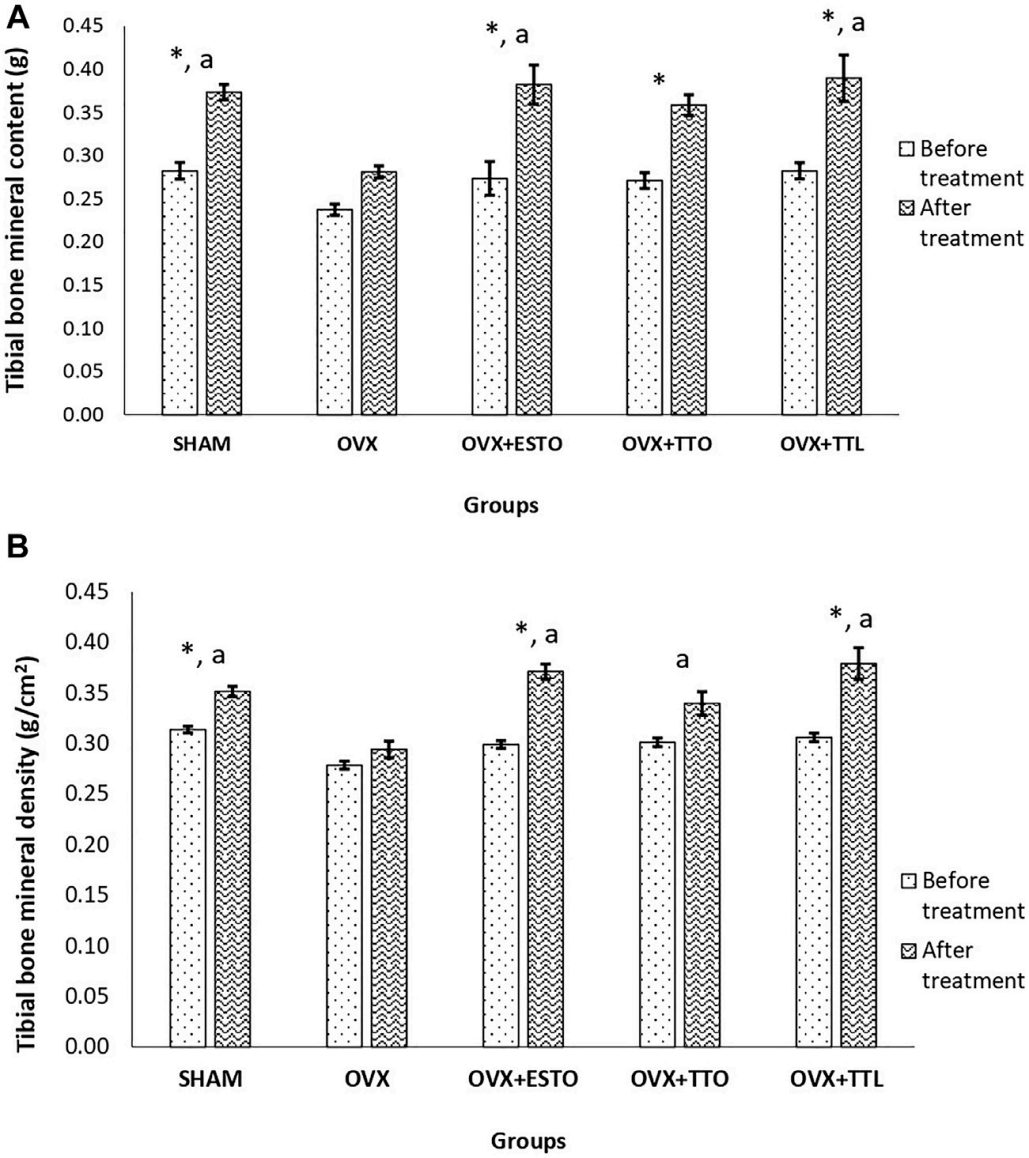
DEXA Parameters (pre- and posttreatment). **(A)** Bone mineral content of the tibial bone. **(B)** Bone mineral density of the tibial bone. SHAM, sham-operated group; OVX, ovariectomized control group; OVX + ESTO, ovariectomized + oral estrogen; OVX + TTO, ovariectomized + daily oral tocotrienol; OVX + TTL, ovariectomized + IO injection of tocotrienol + PLGA. *Significant difference (*p* < 0.05) when compared before and after treatment. ^a^Significant difference (*p* < 0.05) when compared with the ovariectomized control group (OVX). The data were expressed as mean + SEM.

As shown in Figure 4, after 8 weeks of treatment, there was no gain in the tibial bone mineral content of the OVX group compared with the SHAM group. Oral estrogen (OVX + ESTO) was able to increase the tibial bone mineral content by 35.48% compared with the pretreatment content. Both forms of tocotrienol showed similar effects to estrogen, but IO injection of tocotrienol + PLGA (OVX + TTL) seemed to produce a higher percentage increase (34.70%) compared with the oral form (OVX + TTO) of only 30.81%. Similar patterns were seen with the bone mineral density parameter of the tibial bone with oral estrogen showing the highest percentage increase (14.38%), followed by IO injection of tocotrienol + PLGA and oral tocotrienol at 9.43% and 8.75%, respectively.

### Microcomputed Tomography of Tibial Bone

MicroCT is a nondestructive method that provides 3D images of the bone without destroying the sample, permitting inner reconstruction by radiographic image sections. By using µCT, bone quality measurements such as mineral properties and bone microarchitecture could be measured (Irie et al., 2018). Trabecular bone should produce better micro-CT parameters than cortical bone as the changes due to ovariectomy are more dramatic (Osterhoff et al., 2016).

**FIGURE 5:**
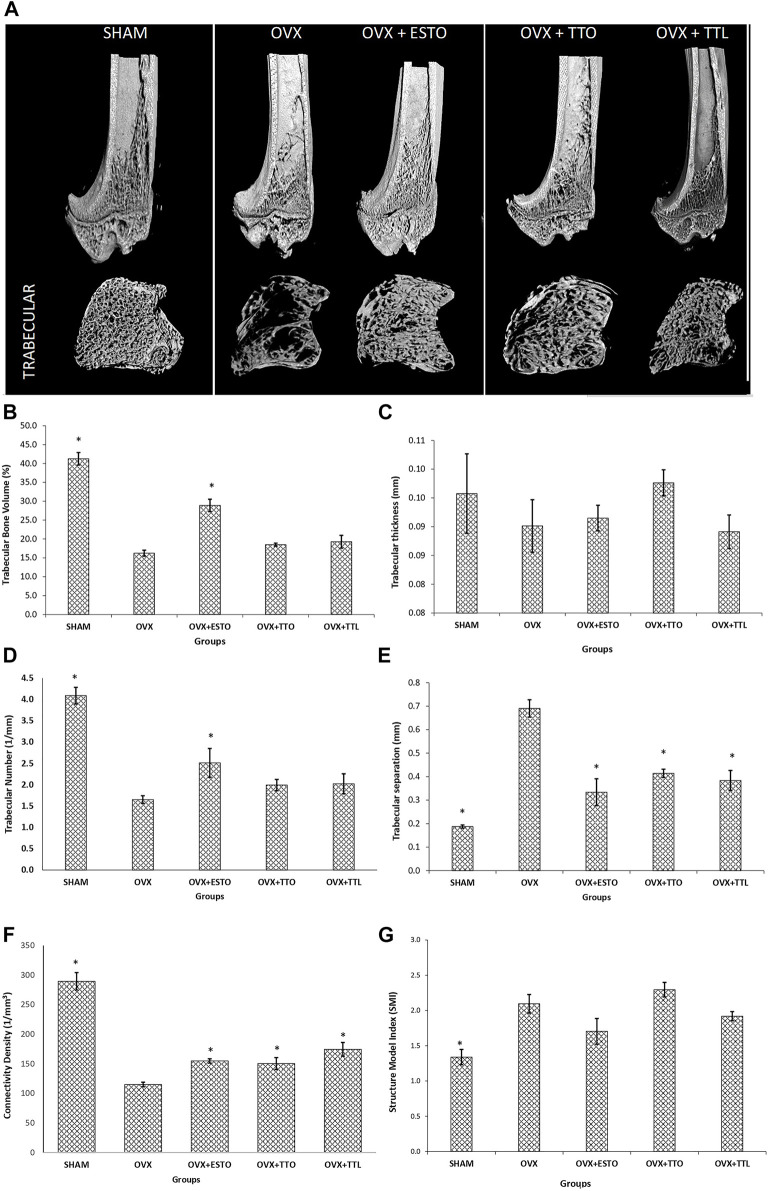
Microcomputed tomography (micro-CT) parameters. **(A)** Three-dimensional (3D) images of tibial trabecular and cortical trabecular bones. **(B)** Trabecular bone volume of the tibial trabecular bone. **(C)** Trabecular thickness of the tibial trabecular bone. **(D)** Trabecular number of the tibial trabecular bone. **(E)** Trabecular separation of the tibial trabecular bone. **(F)** Connectivity density of the tibial trabecular bone. **(G)** Structure model index (SMI) of the tibial trabecular bone. SHAM, sham-operated group; OVX, ovariectomized control group; OVX + ESTO, ovariectomized + oral estrogen; OVX + TTO, ovariectomized + oral tocotrienol; OVX + TTL, ovariectomized + local injection of tocotrienol. *Significant difference (*p* < 0.05) when compared with the ovariectomized control group (OVX). The data are expressed as mean + SEM.

Micro-CT images of the 3D structure of the right tibia formed after micro-CT reconstruction is shown in Figure 5A. Based on the images, the OVX group had the worst deterioration of the trabecular network. Both tocotrienol groups (daily oral and one-time IO injection) showed a more complete trabecular network compared with the OVX group. In terms of the trabecular volume of the tibia, only oral estrogen (OVX + ESTO group) was able to maintain the trabecular volume until it was significantly higher than that of the OVX group and at par with that of the SHAM group. Both forms of tocotrienol failed to emulate the effects of estrogen. As for trabecular thickness, no significant changes were seen in any groups, while for trabecular number, only oral estrogen was able to restore the number of trabeculae affected by ovariectomy. Ovariectomy had caused increased separation between the trabeculae, but the separation was significantly suppressed in all the other groups. In terms of connectivity density, the reductions in this parameter due to ovariectomy were significantly prevented in all the treatment groups. The structure model index (SMI), which can be defined as the deterioration of trabecular or cancellous bone structure due to aging and disease, is characterized by a conversion from “plate-like” to “rod-like.” By measurement of SMI, it is possible to quantify the characteristic form of a three-dimensionally described structure in terms of the number of plates and rod composing the structure. For an ideal plate and rod structure, the SMI value is 0 and 3, respectively, while for a structure with both plates and rods of equal thickness, the value lies between 0 and 3, depending on the volume ratio of rods and plates. The high SMI value indicated the bone deleterious effects of ovariectomy with the conversion of plate to rod-like structure. Only SHAM group had significantly lower SMI value than the OVX group. Similar positive trends were seen for OVX + ESTO and OVX + TTL groups, but they were not significant enough to match those of the SHAM group. All results of the micro-CT parameters are shown in Figures 5B–G.

### Bone Biomechanical Strength

**FIGURE 6:**
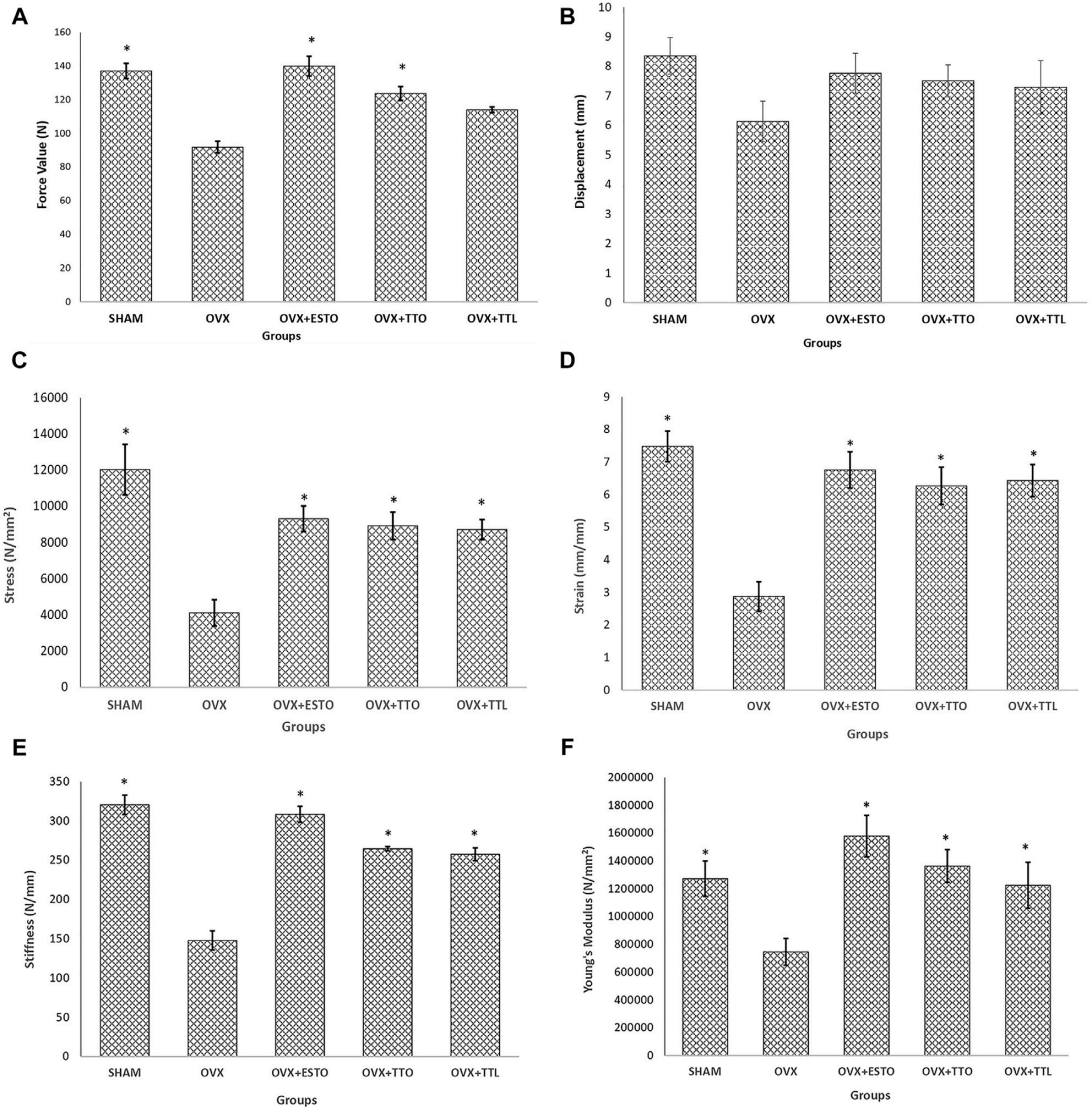
Biomechanical parameters. **(A)** Maximum force before failure of the tibial bone. **(B)** Maximum displacement before failure of the tibial bone. **(C)** Maximum stress before failure of the tibial bone. **(D)** Maximum strain before failure of the tibial bone. **(E)** Stiffness of the tibial bone. **(F)** Young’s modulus of the tibial bone. SHAM, sham-operated group; OVX, ovariectomized control group; OVX + ESTO, ovariectomized + oral estrogen; OVX + TTO, ovariectomized + daily oral tocotrienol; OVX + TTL, ovariectomized + IO injection of tocotrienol + PLGA. *Significant difference (*p* < 0.05) when compared with the ovariectomized control group (OVX). The data are expressed as mean + SEM.

Bone strength is the ultimate parameter to indicate the ability of a bone to resist fracture. There are six main biomechanical bone parameters. In Figures 6A–F, it was shown that for all the parameters, except maximum displacement, estrogen and both forms of tocotrienol were able to reverse the ovariectomy-induced deterioration in bone strength.

## Discussion

Tocotrienols are associated with poor bioavailability due to low binding affinity with hepatic α-tocopherol transfer protein that preferentially selects α-tocopherol (Packer et al., 2001). The poor bioavailability is also related to saturation of Niemann–Pick C1-like 1 transport protein at a high concentration of tocotrienol, thus, limiting its intestinal absorption (Abuasal et al., 2010). This has led to more research on how to overcome the poor oral bioavailability of tocotrienol. Tocotrienol has better antioxidant capacity compared with other forms of vitamin E (Ahsan et al., 2014). Several bone studies have tried combining tocotrienol with drug delivery systems (DDS) to improve its bioavailability, such as polymeric DDS (Ibrahim et al., 2014; Ibrahim et al., 2015) and self-emulsifying DDS (Mohamad et al., 2021). Polymeric DDS helps to deliver therapeutic agents to specific targeted sites in a controlled manner (Sung and Kim, 2020). Drug challenges such as *in vivo* instability, poor bioavailability, poor solubility, poor absorption, and adverse effects could be solved using PDDS (Jahangirian et al., 2017). In PDDS, the polymer acts either as the bioactive polymer (a polymer drug) or more commonly as an inert carrier, in which a drug substance is covalently linked as in polymer–drug conjugates, polymer–protein conjugates, and polymeric micelles (Liechty et al., 2010). PDDS has been applied in biomedical applications to deliver drug substance to the target biological environment (Mhlwatika and Aderibigbe, 2018; Alven and Nqoro, 2020). Specifically for osteoporosis, targeting drugs to specific bones that are at high risk of fracture could be a good option to prevent osteoporosis complications.

In the present study, we compared the bone protective effects of two forms of tocotrienol administered to ovariectomized rats. One was the conventional oral tocotrienol, which require high daily doses to account for its high metabolism and lack of specific transfer protein in the liver. The other form was tocotrienol combined with PLGA, a polymer nanocarrier and injected directly into the bone *via* intraosseous route. Once injected into the targeted bone, tocotrienol particles would be released slowly by the nanocarrier. For real-life application, the tocotrienol–PLGA combination can be injected into the fracture line during surgical fixation of a pathological fracture, before the wound is closed. The benefit and effectiveness of this type of tocotrienol delivery have been demonstrated in an osteoporotic rat model by Ibrahim et al. (2014). The tocotrienol–PLGA combination was able to promote tibial fracture healing in the ovariectomized rat model. However, no comparison was made with the oral form of tocotrienol. Therefore, the present study was conducted to determine whether the tocotrienol–PLGA combination could produce the same beneficial effects on an intact bone of ovariectomized rat model. The one-time local bone injection of tocotrienol–PLGA combination was also compared with the daily oral intake of tocotrienol.

As in previous studies, ovariectomy has led to an increased bone turnover in the ovariectomized control group (Kennedy et al., 2009; Yoon et al., 2012). This was demonstrated by the high osteocalcin and CTX levels recorded at the end of the treatment. As expected, administration of estrogen was able to suppress the high bone turnover conditions, causing a marked reduction in bone formation and resorption markers. Both forms of tocotrienol, either daily oral tocotrienol or one-time local injection of tocotrienol–PLGA combination, were able to emulate the actions of estrogen. This has proven that both forms of tocotrienol were as effective as estrogen in keeping the bone turnover in check for the postmenopausal osteoporosis rat model. Our results for osteocalcin and CTx levels were not parallel to that of Mohamad et al. (2018), whereby administration of annatto tocotrienol at 60 and 100 mg/kg to buserelin-induced osteoporosis rat model for 3 months was unable to improve the bone biochemical parameter. However, the tocotrienol was given orally, and the results may be different if it were given *via* the drug delivery system (PLGA) as used in our study. Several mechanisms were suggested for the ability of tocotrienol to protect against ovariectomy-induced bone loss such as antioxidant (Soelaiman et al., 2012; Muhammad et al., 2013), anti-inflammatory (Soelaiman et al., 2012), and inhibition of hydroxy-methyl-glutaryl-coenzyme A (HMG-CoA) reductase in mevalonate pathway (Abdul-Majeed et al., 2012; Deng et al., 2014). Tocotrienol suppressed the mevalonate pathway via degradation of the HMG-CoA reductase protein that catalyzes the conversion of HMGCoA to mevalonic acid in the cholesterol synthesis pathway, thus, causing reductions in prenylated proteins (Parker et al., 1993; Song and DeBose-Boyd, 2006). The inhibition of prenylated proteins, which include RhoA and Rac1, led to the stimulation of bone morphogenetic proteins (BMP)-2 (Hagihara et al., 2011; Onishi et al., 2013). The activation of BMP signaling pathways by BMP-2 could promote osteoblastic differentiation in osteoporotic rats (Zhao et al., 2019). Moreover, in an *in vitro* study, annatto tocotrienol was demonstrated to activate the osteogenic activity of preosteoblastic MC3T3-E1 cell line via inhibition of RhoA and promotion of BMP-2 protein (Wan Hasan et al., 2018).

The deleterious effects of ovariectomy and the countering effects of estrogen were demonstrated in the DEXA parameters of the tibial bone. The ability of tocotrienol–PLGA combination to protect against ovariectomy-induced bone loss may be contributed to its intraosseous administration to the tibiae. The nanocarrier, PLGA, held on to the tocotrienol particles and slowly releasing them throughout the study. With the tibial bone receiving maximum and constant tocotrienol particles, it was protected against the deleterious effects of ovariectomy on bone mineral content and density. This was supported by the findings of Mohamad et al. (2021) that the bone mineral content of ovariectomized rats receiving tocotrienol via self-emulsifying DDS was significantly higher than that of the control group. This indicated that tocotrienol given orally via the drug delivery system was effective in protection against ovariectomy-induced bone loss. It is noteworthy that the self-emulsifying DDS of tocotrienol was delivered orally every day for a duration of 2 months. As for our study, the tocotrienol–PLGA combination was delivered directly to the targeted bone via intraosseous injection, and it was administered only once.

Micro-CT has produced detailed microarchitectural changes in the tibiae of the rat model. Estrogen was able to protect the trabecular volume and trabecular number parameters of the tibiae against ovariectomy-induced changes, but both forms of tocotrienols failed to do the same. However, both forms of tocotrienols were as effective as estrogen in protecting the trabecular separation and connectivity density parameters. Trabecular separation and connectivity density are important assessments for bone microarchitecture and microarchitectural complexity, respectively. Based on the micro-CT parameters, both forms of tocotrienol were equally effective in protecting the bone against ovariectomy-induced changes.

Computational tools to interpret bone stiffness and potential for mechanical failure or fracture could provide further insight into the effects of certain agents on osteoporotic bone (Wu et al., 2015). Biomechanical strength testing is a numerical method and finite element in analyzing bone stiffness. Bone biomechanical testing is the most important bone parameter as it can be used to assess protection offered by test agents against pathological fractures, the main complication of osteoporosis (Chen et al., 2016). Both forms of tocotrienols were equally effective to estrogen and with each other in strengthening the tibiae of ovariectomized rats, therefore, protecting the bone against fractures. Mohamad et al. (2012) reported that the fracture callus of a rat group receiving oral palm tocotrienol-enriched fraction had significantly higher stress parameter than the control group (Mohamad et al., 2012). Meanwhile, Ibrahim et al. (2014) found that a single injection of annatto tocotrienol combined with PLGA, locally delivered to the muscles near the fracture site, had significantly higher callus strength in terms of the load and stress parameters compared with control (Ibrahim et al., 2014). These studies may have indicated that both forms of tocotrienol, the oral form or in combination with PLGA, were effective for fracture healing and for the bone health in general.

### Limitations

The need of bone drilling to provide an access for intraosseous injection is considered an invasive procedure. Therefore, for clinical application, tocotrienol may be administered to the already fractured osteoporotic bone during orthopedic surgery.

### Future Perspectives

Local injection of the tocotrienol–PLGA combination has demonstrated good potential for use in bone health. Only a single IO injection was required to produce similar bone protective effect to estrogen and oral daily tocotrienol administrations. For potential clinical use, IO injection of tocotrienol can be administered during surgical fixation of bone fracture, as the tocotrienol–PLGA combination can be conveniently injected into the fracture line before closing the surgical wound. Further studies on the evaluation of the drilling site healing with respect to tocotrienol–PLGA could be performed to predict fracture risk and assess fracture healing.

## Conclusion

One-time IO injection of the tocotrienol–PLGA combination was as effective as daily oral tocotrienol in attenuating ovariectomy-induced bone changes. The tocotrienol–PLGA combination has the advantages of requiring lower dose and frequency of administration as tocotrienol was released in a controlled and consistent manner into the bone microenvironment.

## Data Availability

The original contributions presented in the study are included in the article/Supplementary Material. Further inquiries can be directed to the corresponding authors.
